# Maternal-fetal conflict and the timing of birth

**DOI:** 10.1093/emph/eoaf023

**Published:** 2025-09-12

**Authors:** Andrew I Furness

**Affiliations:** Independent Researcher, Gainesville, FL, USA

**Keywords:** birthweight, gestation length, parent-offspring conflict, parturition, pregnancy, preterm birth

## Abstract

Evolutionary theory predicts mammalian offspring will favor greater investment than parents are willing to provide, leading to conflict over resource transfer. This theory of parent-offspring conflict has been applied to resource transfer across the placenta. Birthweight and gestation length are functionally linked, suggesting that the timing of birth might also represent a focal point of maternal-fetal conflict. This hypothesis relies on two assumptions: mother and offspring have different fitness optima and both parties exert partial control over the timing of birth. It is argued, and evidence is reviewed, that suggests offspring benefit from slightly longer gestation than the maternal optimum, and that fetal and maternal genes both influence gestation length. We might therefore expect an evolutionary history of maternal-fetal conflict over the control of parturition. Evidence consistent with this hypothesis includes the effect of imprinted genes, as revealed through imprinting disorders, on gestation length; the wide variability in parturition mechanism(s) among mammalian species; and the complex physiology of human parturition including initiation by multiple different pathways with inputs from both mother and offspring. One potential consequence of a history of maternal-fetal conflict over control of the mechanisms of parturition is that the timing of birth may be subject to a greater degree of dysregulation than simpler physiological systems subject to single-party control.

“*Parturition itself has considerable risks for both mother and infant. At birth, the fetal cranium is close to the maximum size that can be delivered through the maternal pelvic outlet. An important evolutionary question is why the fetus waits so long before it is born. I suspect that fetuses attempt to remain in the womb until the nutritional benefits of remaining inside are not worth the increasing risks of delivery. Over evolutionary time, the duration of pregnancy is possibly determined by a conflict between fetal genes favoring slightly longer gestations and maternal genes favoring slightly shorter gestations. This question deserves further study.*”[1]

## INTRODUCTION

Mammalian offspring are predicted to favor greater parental investment than parents are willing to provide, leading to conflict over the transfer of resources [2]. In the original presentation of this idea—that of parent-offspring conflict—Trivers [2] focused on overt behavioral manifestations of conflict between mother and offspring around the time of weaning while explicitly realizing that the same principles are likely at play in other arenas: “It is possible, for example, that weight at birth in a mammal such as humans is strongly associated with the offspring’s survival in subsequent weeks, but that the cost to the mother of bearing a large offspring is considerably greater than some of her ensuing investment. In such circumstances, conflict prior to birth over offspring’s weight at birth may be more intense than conflict over nursing in the weeks after birth.”

Approximately two decades later, a compelling case was made for maternal-fetal conflict during pregnancy [1]. Specifically, Haig [1] interpreted several otherwise puzzling aspects of human pregnancy as byproducts of underlying conflict over resource transfer. For example, the fetal-placental unit produces and releases large quantities of growth-promoting hormones directly into the maternal bloodstream, where they bind to maternal receptors. Yet, the action of these offspring-derived hormones appear to be counteracted by maternal tissues and ultimately have minimal effect on maternal physiology. This *over*-production of growth-promoting hormones and evolved maternal insensitivity to their effects is consistent with an arms race over the control of offspring resource provisioning. Additional evidence for parent-offspring conflict over *in utero* resource transfer has been broadly reviewed [3–7], particularly with respect to the phenomena of genomic imprinting [8–11].

Another offspring strategy to extract additional maternal investment may be to extend gestation. This assumes that offspring benefit from staying *in utero* longer than is optimal for the mother, and both parties—mother and offspring—exert influence over the timing of birth. In this view, conflict over the timing of birth (i.e. parturition) may be somewhat analogous to conflict over when to end nursing (i.e. weaning), except here the mechanisms would likely be physiological rather than behavioral. This idea has been mentioned in passing [1, 12, 13] but has not received a detailed treatment. Here, I further develop and discuss this hypothesis and then review available supporting evidence.

## MOTHER AND OFFSPRING FITNESS OPTIMA FOR GESTATION LENGTH

**Figure 1:**
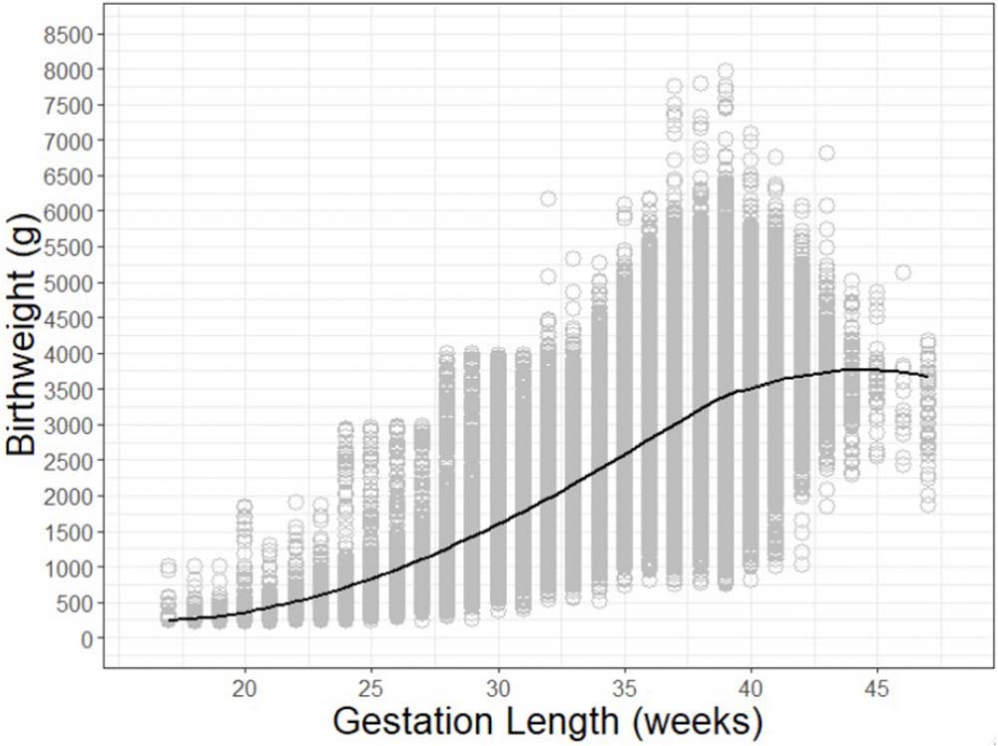
A plot of gestation length and birthweight for all singleton live births in the USA in 2022 (*n* = 3 553 730). The black line has been fit with a locally estimated scatterplot smoothing function. Data source: USA vital statistics natality birth data from the National Bureau of Economic Research (https://www.nber.org/research/data/vital-statistics-natality-birth-data).

**Figure 2:**
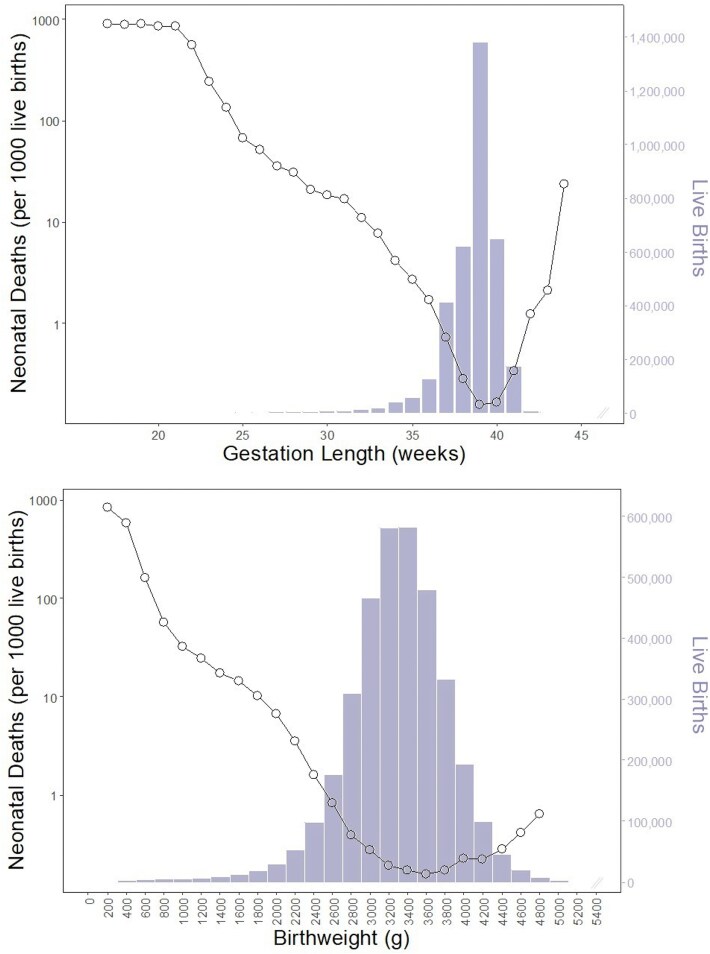
Neonatal mortality rate as a function of (a) gestation length and (b) birthweight for all singleton live births in the USA in 2022. (a) Mortality is lowest at 39 to 40 weeks gestation. The mean gestation length in this population is 38.45 weeks and the median is 39 weeks (*n* = 3 551 145). (b) Mortality is lowest within the birthweight increment of 3500 to 3700 g. The mean birthweight in this population is 3272 g and the median is 3300 g (*n* = 3 551 074). Note, the right tail of both distributions has been visually truncated due to very low sample size. Gestation length refers to the obstetric estimate of gestation. Data source: USA vital statistics natality birth data from the National Bureau of Economic Research (https://www.nber.org/research/data/vital-statistics-natality-birth-data).

The hypothesis of maternal-fetal conflict over the timing of birth assumes that offspring gain fitness benefits from lengthening gestation beyond the maternal optimum. It is perhaps easiest to envision such benefit if offspring weight increases as a function of gestation length (Fig. 1), and survival is positively correlated with birthweight (Fig. 2). In humans this appears to be the case. Furthermore, it has long been recognized that the birthweight at which offspring mortality is minimized is greater than the mean birthweight in a population [14–17]. This presents a conundrum: “It is natural to assume that in consequence of the action of natural selection the mean value of any biological measurement would be the most normal value and associated with the most favourable survival rate. In the case of birth weight the optimal value from the point of view of avoiding stillbirth or neonatal mortality is clearly greater than the mean” [14]. One subsequent suggestion is that this discrepancy between expectation and reality may be explained by maternal-fetal conflict [18]; the optimum birthweight from the perspective of maximizing lifetime maternal reproductive success is predicted to be lower than that which maximizes individual offspring survival, with the observed birthweight (and population mean value) representing a compromise between the maternal and offspring optima.

The same principle could potentially be true for gestation length. Here, I examined neonatal mortality as a function of birthweight and gestation length for all singleton live births in the USA in 2022 (Fig. 2). Consistent with prior work, I found the mean birthweight in this population (3272 g) to be lower than the birthweight increment in which offspring mortality was minimized (3500 to 3700 g) (*n* = 3 551 074). A similar pattern was observed with respect to gestation length. The mean gestation length in this population was 38.45 weeks, while neonatal mortality was lowest at 39 to 40 weeks gestation (*n* = 3 551 145). In humans there are a number of offspring benefits to longer gestation length, even among term pregnancies (i.e. those between 37 and 41 weeks gestation), including better cognitive and motor development [19–21].

In the last few weeks of pregnancy, offspring gain substantial weight, exhibit rapid brain growth, and accumulate large amounts of subcutaneous fat [1, 22–24]. This rapid growth comes at a cost; during this period, maternal maximum sustained metabolic rate approaches what is thought to be a metabolic ceiling [25] and total energy expenditure peaks [26]. Conversely, the cost of nursing—although *overall* more energetically costly than pregnancy [26, 27]—is presumably lower just after birth than at any point in the following six months of exclusive nursing [22, 25]. Furthermore, it has been argued that fat can be more efficiently transferred to offspring through breast milk than across the placenta [22, 24], lactation can be more readily supported by mobilizing maternal fat stores [22, 24, 27], mean milk production is approximately the same between mothers from developed and developing countries [26], and mothers are in greater control of nursing rate than resource transfer across the placenta [13]. Finally, very large size at birth increases the likelihood of birth complications such as shoulder dystocia [28, 29]. Together, these considerations suggest initiating birth slightly earlier than is optimal for offspring may be a way in which mothers reduce the risk of birth complications and health costs associated with large size at birth, and/or mitigate the energetic costs of late pregnancy as they approach their metabolic limit [25].

**Figure 3:**
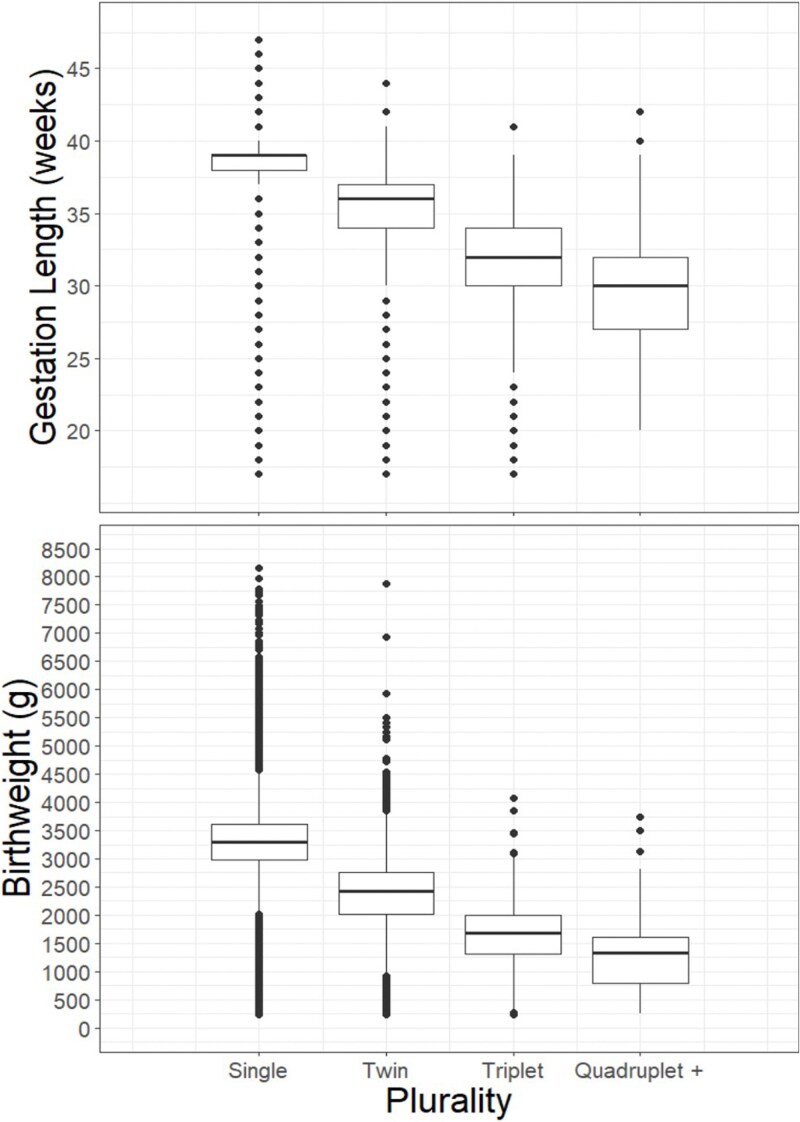
Gestation length (a) and birthweight (b) as a function of plurality for all live births in the USA in 2022 (*n* = 3 673 224 for gestation length, *n* = 3 672 945 for birthweight). Preterm birth (i.e. before 37 weeks gestation) occurred in 8.7% of singleton births, 61.3% of twins, 99.0% of triplets, and 95.9% of quadruplets and higher. Shorter gestation lengths were accompanied by a corresponding decrease in birthweight (median birthweight for singletons: 3300 g, twins: 2420 g, triplets: 1680 g, and quadruplets and higher: 1335 g). Data source: USA vital statistics natality birth data from the National Bureau of Economic Research (https://www.nber.org/research/data/vital-statistics-natality-birth-data).

The width of the birth canal places a hard-bound constraint on fetal size at birth [30], but in some circumstances birth occurs well before this point is reached. This is illustrated with multiple births (Fig. 3). Both gestation length and birthweight decrease in lockstep as the number of births increases (i.e. from singletons, to twins, to triplets, to quadruplets). In a somewhat similar vein, among singleton pregnancies, the likelihood of preterm birth (i.e. before 37 weeks of completed gestation) increases when gestating male offspring [31], with low pre-pregnancy maternal body mass index (BMI) [32], and following a short interbirth interval [33]. These observations suggest energetics play a role in the timing of birth. The metabolic crossover hypothesis posits that the signaling cascade that initiates labor begins with the inability of the mother to meet offspring metabolic requirements, or when there is a “cross-over in the curves reflecting fetal metabolic requirements and maternal ability to meet those requirements” [24]. Maternal-fetal conflict over the timing of birth is congruent with the metabolic crossover hypothesis and pelvic constraints on fetal size at birth, but adds an additional detail. Specifically, it predicts offspring accrue fitness benefit from pushing mother’s nearer to their metabolic maxima, or edging closer to the physical size constraint determined by pelvis size. Nonetheless, by logical and physical necessity, there will be some point (an upper bound) when staying *in utero* too long has negative fitness consequences for both offspring and mother [34]. Thus, there is a natural cap to the extent of conflict. Given an uncomplicated term pregnancy, we might therefore expect conflict over gestation length to be confined to a relatively narrow period late in pregnancy. We also might anticipate the threshold for initiation of labor to be lower for mother than offspring, particularly when maternal health is at risk.

## GESTATION LENGTH AS A SHARED TRAIT

For conflict to be realized and have phenotypic effects, both parties must be able to exert influence over the timing of birth. This leads to the question of who controls gestation length—is this a trait determined by the mother, the offspring, or both in combination? This question has been addressed using several approaches (Table 1).

**Table 1:** Evidence for maternal genetic (a) and fetal genetic (b) contributions to gestation length.

**(a) Evidence that maternal genotype influences gestation length**	**Reference(s)**
Quantitative genetic analysis that partitions variation in gestation length into genetic and environmental components indicates maternal genetic contribution	[45, 48, 97]
Significant positive correlation between mother’s own gestational age and her offspring	[35–37]
Genetic influence on preterm birth, when mother considered proband ^a^	
Risk of preterm delivery higher in women who were born preterm	[39, 40]
Prior preterm delivery is risk factor for subsequent preterm delivery	[41, 42]
Recurrent preterm delivery most likely in same week as prior preterm delivery (i.e. gestation length consistent across pregnancies in same mother)	[38]
Family history of preterm birth (e.g. sisters share preterm delivery risk)	[43, 44, 53, 93]
Heritability of preterm birth/gestation length	[37, 43, 98, 99]
Genome-wide association/candidate gene association studies indicate association between certain maternal loci and preterm birth	[49–52]
Physiological details of parturition	
Molecules involved in initiation of parturition produced by mother (e.g. corticotropin-releasing hormone, stress inflammatory cytokines)	[72]
**(b) Evidence that fetal genotype influences gestation length**	**Reference(s)**
Quantitative genetic analysis that partitions variation in gestation length into genetic and environmental components indicates fetal genetic contribution	[45, 48, 97]
Significant positive correlation between father’s own gestational age and his offspring	[36, 37]
Paternity affects preterm birth risk as indicated through partner change (father) situations	[42, 46]
Offspring genetics/genetic disorders affect gestation length	
Fetal sex influences gestation length and likelihood of preterm birth	[31, 100–103]
Fetal imprinting disorders influence gestation length (e.g. Beckwith–Wiedemann syndrome)	see Table 2
Fetal genetic disorders influence gestation length (e.g. Ehlers–Danlos syndrome)	[47]
Genome-wide association/candidate gene association studies indicate association between certain fetal loci and preterm birth	[49–52]
Physiological details of parturition	
Molecules involved in pregnancy maintenance produced by fetal-placental unit (e.g. progesterone)	[72]
Molecules involved in initiation of parturition produced by fetal-placental unit (e.g. corticotropin-releasing hormone, stress inflammatory cytokines, and estradiol)	[72]

a Consistent with maternal genetic contribution, but not all listed references distinguish between maternal and fetal genetic effects.

Maternal genetic effects on gestation length are intuitive and well supported. A significant positive correlation has been found between a mother’s own gestational age and that of her offspring [35–37]. Furthermore, gestation length is consistent across pregnancies in the same mother [38]. Finally, the risk of preterm delivery is higher in women who were born preterm [39, 40], prior preterm delivery is a risk factor for subsequent preterm delivery [41, 42], and preterm birth runs in families—with, for example, sisters sharing preterm delivery risk [43, 44]. This data suggests a genetic influence on preterm birth, when the mother is considered the proband [45].

**Figure 4:**
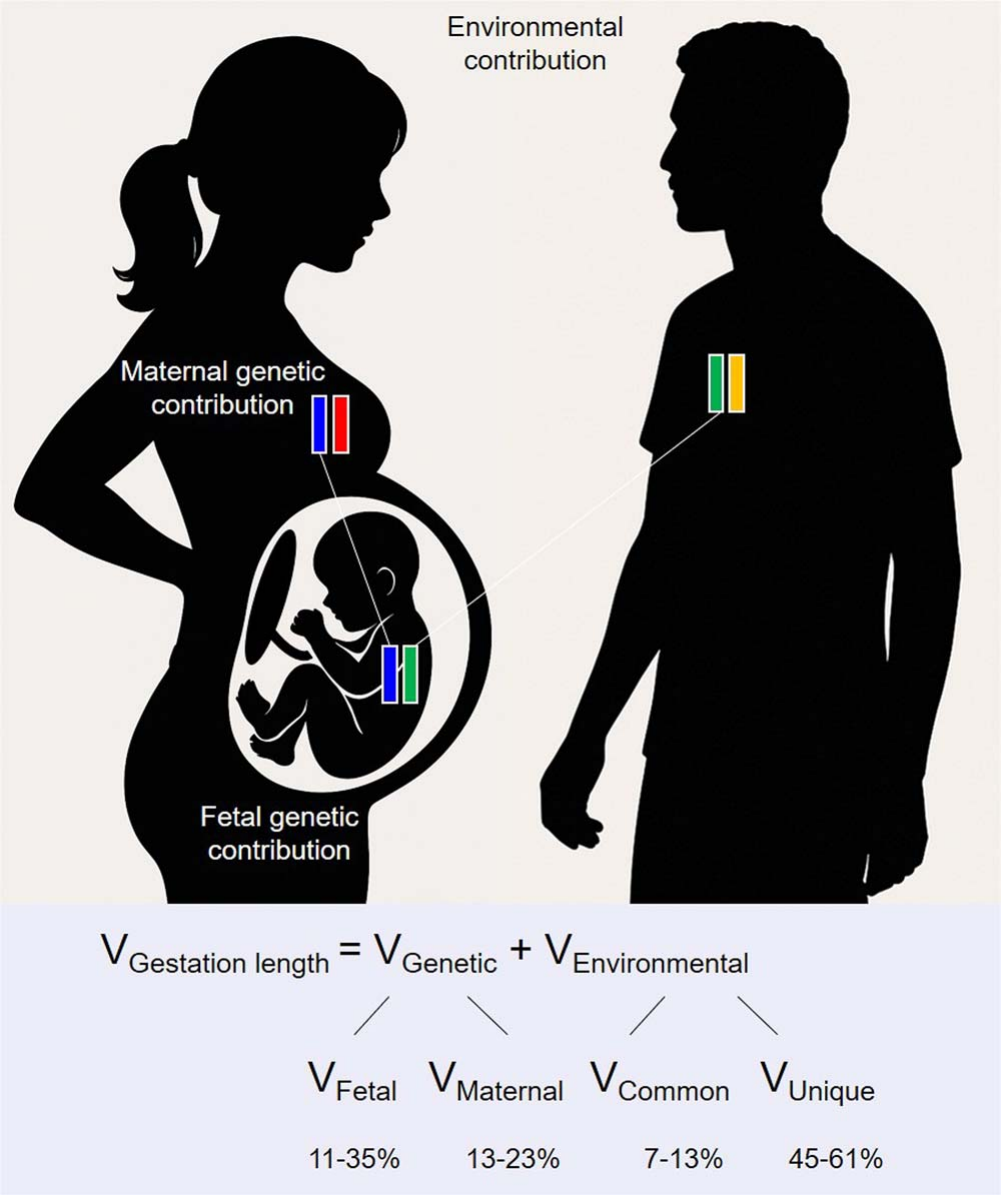
Phenotypic variation in gestation length can be attributed to either genetic (V_Genetic_) or environmental (V_Environmental_) factors. With gestation length (or its dichotomous analog, preterm birth), either mother or offspring could reasonably be considered the proband, and evidence suggests both influence this trait (Table 1). Therefore, the genetic component can be further decomposed into fetal genetic (V_Fetal_: represented by maternally inherited blue allele and paternally inherited green allele) and maternal genetic (V_Maternal_: represented by maternal blue and red alleles) contributions. Likewise, the environmental component can be subdivided into contributions common to all births by the same mother (V_Common_) and those that are unique to each pregnancy (V_Unique_). Quantitative genetic studies of gestation length in families consisting of the offspring of twins, full siblings, half-siblings, and cousins demonstrate both fetal (11%–35%) and maternal (13%–23%) genetic contributions to the timing of birth, with the largest contribution attributed to environmental factors [48].

There is also evidence for fetal genetic effects on gestation length. Much of this comes by way of demonstration that the paternal genome influences gestation length. A significant correlation has been found between a father’s own gestational age and that of his offspring [36, 37]. Paternity affects preterm birth risk [42, 46]. For example, Li [42] addressed the question: If a child is born preterm, does changing partners affect the likelihood of preterm birth in the subsequent pregnancy? Women were divided into 3 cohorts based upon the gestational age of the first pregnancy: early preterm, preterm, and normal. Among women in the early preterm cohort, changing partners reduced the risk of early preterm delivery by 33% in the subsequent pregnancy relative to women who did not change partners. In contrast, among women in the normal cohort, changing partners increased the risk of early preterm delivery by 16% in the subsequent pregnancy. This is consistent with a paternal genetic effect on the likelihood of preterm birth (i.e. gestation length). The paternal genetic contribution is undoubtedly brought about through the offspring’s effect on gestation length, as offspring inherit 50% of genes from their father (Fig. 4). Finally, genetically determined traits (such as fetal sex) and fetal genetic disorders (such as Ehlers–Danlos syndrome) affect gestation length and the likelihood of preterm birth [31, 47].

Some of the strongest evidence for both maternal and fetal genetic contributions to the timing of birth comes from quantitative genetic studies that partition phenotypic variance in gestation length into its genetic and environmental components. The use of sophisticated study designs that employ family pedigrees allows genetic effects to be further partitioned into fetal genetic and maternal genetic contributions (Fig. 4). A review of such studies found both fetal (11%–35%) and maternal (13%–23%) genetic contributions to the timing of birth, with the largest contribution attributed to environmental factors [48]. York et al. [48] conclude that “improvements in biometric-genetic studies of twin kinships, half-sibships, and cousinships have now demonstrated a sizeable fetal genetic and maternal genetic contribution to the spontaneous onset of labor. This is an important development because previous literature for the most part reports only an influence of the maternal genome.”

Candidate and genome-wide association studies have linked preterm birth with specific genetic loci found in mother and fetus [49–52]. This supports the conclusion from quantitative genetic studies of both maternal and fetal contributions to gestation length [48]. These genetic variants associated with preterm birth are of small effect [53–55], and appear to have diverse functions [54–56], including several involved in inflammatory and immune pathways [49, 51, 53, 57]. Further study has shown that genomic regions associated with preterm birth harbor diverse evolutionary signatures, including negative selection (i.e. sequence conservation), population differentiation (i.e. local adaptation), positive selection (i.e. accelerated substitution rate), and balancing selection (i.e. balanced polymorphism) [58]. Finally, with respect to gestation length and birthweight, a complex relationship has been found between maternal and fetal genomes, including antagonistic pleiotropic effects [52]. Some of the above evolutionary patterns—in particular positive selection, population differentiation, and antagonistic pleiotropy—might be consistent with a history of maternal-fetal conflict, in addition to other evolutionary processes mentioned by the authors, such as local adaptation or coadaptation between maternal and fetal effects.

Box. Relationship between maternal-fetal conflict and other related hypotheses

Several different hypotheses have been developed to account for certain features of human childbirth (e.g. cliff-edge model of obstetric selection, obstetric dilemma hypothesis, metabolic crossover hypothesis). However, none of these hypotheses can be viewed as strict alternatives to that of maternal-fetal conflict. We would expect maternal-fetal conflict over the timing of birth whenever two underlying assumptions are met: divergent fitness optima between mother and fetus, and both parties influence gestation length. As such, the hypothesis of maternal-fetal conflict cuts across these other hypotheses. Whatever explanatory value they hold, they do not—at least as originally formulated—explicitly take into account the predicted divergent fitness optima of mother and offspring. Therefore, the hypothesis of maternal-fetal conflict can be thought of as adding an important additional detail. Specifically, offspring obtain fitness benefit from edging closer to the physical size constraint determined by maternal pelvis size or pushing mother’s nearer to their metabolic maxima than is in their mother’s own best interests. This is predicted to create the conditions for maternal-fetal conflict over the control of parturition—ultimately, leaving evolutionary signatures of conflict in the genome and in the physiological details of parturition, as well as having downstream phenotypic consequences (see main text for details).

**Table:** 

**Hypothesis**	**Description/assumptions**	**Predicted outcome**	**Reference**
Cliff-edge model of obstetric selection	Mismatch between a wide symmetric phenotype distribution (i.e. neonatal size versus pelvic canal width) and an asymmetric cliff-edged fitness function	High incidence of obstructed labor	[59]
Obstetric dilemma hypothesis	Female pelvis design is shaped by the competing demands of childbirth and bipedal locomotion	Altriciality and relatively difficult childbirth	[60, 61]
Metabolic crossover hypothesis	Gestation length, neonatal size, and altriciality are the consequences of maternal metabolic constraints	Signaling cascade that initiates labor begins with the inability of the mother to meet offspring metabolic requirements	[24, 25]
Maternal-fetal conflict hypothesis	Offspring benefit from slightly longer gestation than is optimal for the mother, and both parties influence gestation length	Evolutionary history of maternal-fetal conflict over control of parturition	This paper

## GENOMIC IMPRINTING AND GESTATION LENGTH

The effect of imprinted genes on gestation length, as demonstrated in imprinting disorders (Table 2), is noteworthy given their purported role in parental conflict over fetal resource transfer [62]. Genomic imprinting refers to an epigenetic phenomenon in which certain genes (~120 in humans) are either expressed or silenced depending upon which parent they are inherited from [6, 8]. In imprinting disorders, this monoallelic expression is disrupted, for example, due to the inheritance of two copies of a chromosome from one parent—referred to as uniparental disomy—or due to deletion of either the maternally inherited or paternally inherited imprinted gene cluster [63, 64]. This results in either both copies being expressed (i.e. double expression) or neither being expressed (i.e. no expression). Imprinting disorders tend to be clinically diagnosed by aberrant fetal and childhood growth phenotypes, and a large number of imprinted genes are involved in fetal growth, including those that underlie these disorders [6, 63]. Here a particular pattern is evident: paternally expressed genes tend to be growth-promoting (and maternally silenced), while maternally expressed genes tend to be growth-suppressing (and paternally silenced) [6]. Furthermore, evidence suggests the paired imprinting disorders of Beckwith–Wiedemann and Silver–Russell syndrome, Angelman and Prader–Willi syndrome, and Kagami–Ogata and Temple syndrome represent genetically and clinically opposite disorders [1, 63–66]. That is, each of these three pairs of imprinting disorders with generally opposing phenotypes (i.e. fetal overgrowth vs. growth retardation) traces back to the same chromosome region and results from either excess paternal expression (and deficient maternal expression) or excess maternal expression (and deficient paternal expression). These patterns have been interpreted in terms of evolutionary conflict, with paternally inherited genes selected to favor greater maternal investment in offspring and maternally inherited genes greater restraint [9, 10, 13, 62]. According to the “parental conflict” theory of genomic imprinting, the observed parent-of-origin expression patterns are the end result of a conflict-driven arms race between maternal and paternal genes over the level of offspring provisioning [8–11]. Imprinting disorders play a crucial role in supporting this theory, with the opposing growth phenotypes interpreted as favoring one parent’s interests over the other, but taken to pathological extremes [13, 65].

**Table 2:** The effect of various imprinting disorders on gestation length.

**Imprinting disorder**	**Chromosome region**	**Epigenetic characterization**	**Fetal growth/birthweight**	**Gestation length**	**Reference(s)**
Beckwith–Wiedemann syndrome	11p15	Excess paternal expression, or deficient maternal expression (e.g. duplication of paternal 11p15, gain of methylation in 11p15 imprinting control region 1)	Overgrowth/fetal macrosomia	Preterm birth rate elevated (39.4%)	[63, 104, 105]
Silver–Russell syndrome	11p15^a^	Excess maternal expression, or deficient paternal expression (e.g. duplication of maternal 11p15, loss of methylation in 11p15 imprinting control region 1)	Growth retardation	2–3 weeks shorter (median 37 weeks), preterm birth rate elevated (41%)	[63, 106, 107]
Angelman syndrome	15q11.2–q13	Excess paternal expression, or deficient maternal expression (e.g. paternal uniparental disomy 15, deletion of maternal 15q11.2-q13)	Normal/no specific information	Normal/no specific information	[108, 109]
Prader–Willi syndrome	15q11.2–q13	Excess maternal expression, or deficient paternal expression (e.g. maternal uniparental disomy 15, deletion of paternal 15q11.2-q13)	Mild growth retardation/low birthweight (30%)	Preterm and post-term birth rate elevated (50% of births 2 weeks earlier or later than due date)	[110–112]
Kagami–Ogata syndrome	14q32	Excess paternal expression, or deficient maternal expression (e.g. paternal uniparental disomy 14, epimutations/deletions of maternal 14q32)	Overgrowth/fetal macrosomia	Preterm birth rate elevated	[64]
Temple syndrome	14q32	Excess maternal expression, or deficient paternal expression (e.g. maternal uniparental disomy 14, epimutations/deletions of paternal 14q32)	Growth retardation/low birthweight (88%)	1–2 weeks shorter (median 38 weeks), preterm birth rate elevated (15%)	[64, 106, 113]

a Imprinted genes on chromosome 7 also likely involved in etiology of Silver–Russell syndrome [63].

Four imprinting disorders are associated with a shortened gestation length and/or elevated rate of preterm birth (Beckwith–Wiedemann syndrome, Silver–Russell syndrome, Kagami–Ogata syndrome, and Temple syndrome), one with a substantially elevated rate of preterm and post-term birth (Prader–Willi syndrome), and one with no apparent effect (Angelman syndrome) (Table 2). The bias toward preterm birth observed in imprinting disorders may seem counterintuitive, as we might expect paternal over-expression to be associated with longer gestation, consistent with greater resource extraction. Indeed, imprinting disorders with excess paternal expression are often characterized by fetal overgrowth (Table 2); however, this may actually make preterm birth more likely (even necessary), given the functional link between fetal growth and timing of birth. Essentially, if a fetus is growing abnormally large, then an early birth may be required because if birth were delayed until term, it would increase the probability of birth complications and even death for both the fetus and mother. Thus, the overall preterm birth bias may become interpretable: imprinting disorders with maternal over-expression may be biased toward preterm birth as this tends to benefit the mother (although taken to pathological extremes), and imprinting disorders with paternal over-expression tend to be characterized by preterm birth given the frequency of fetal overgrowth and its functional link with birth timing. More generally, the fact that gestation length is affected in many imprinting disorders, and by implication influenced by imprinted genes, suggests a history of conflict over the timing of birth—or a history of conflict over fetal resource transfer of which the timing of birth appears to be part and parcel.

An alternative means to examine a potential role for imprinted genes in determining gestation length is to identify genes that influence gestation length through genome-wide association studies and then compare them to those that are known to be imprinted in humans. Using genome-wide meta-analysis (*n* = 195 555), Solé-Navais et al. [52] found 22 loci (24 genetic variants) significantly associated with gestational duration. An analysis of inheritance patterns revealed 15 of these genetic variants influenced gestation through the maternal genome (i.e. maternal genetic effect), 7 through the maternal and fetal genomes, and 2 through the fetal genome alone (i.e. fetal genetic effect). None of the 22 identified loci are known to be imprinted in humans (https://www.geneimprint.com/site/genes-by-species). However, intriguingly, one of the two alleles of fetal-only effect (EEFSEC) was associated with shortened gestation only when maternally transmitted (i.e. it exhibited a parent-of-origin effect, although with low certainty). Furthermore, of the seven loci with both maternal and fetal effects on gestational duration, five exhibited opposite effect direction. Specifically, two alleles (FAF1 and FBXO32) were associated with longer gestation when present in the mother and when maternally transmitted, but shorter gestation when paternally transmitted. Conversely, three alleles (KDR, RAP2C, MYOCD) were associated with shorter gestation when present in the mother and when maternally transmitted, but longer gestation when paternally transmitted. The same allele having opposite effect depending upon whether maternally or paternally transmitted is consistent with a parent-of-origin effect, and perhaps suggestive of underlying parental conflict over fetal gestation length.

## PARTURITION

Recent reviews on the initiation of human parturition have described the process as remarkably elusive [67], obscure [68], unclear [69], not yet fully clarified [70], perplexing to comprehend [71], and “one of biology’s great unsolved mysteries” [72]. Despite being a universal feature of mammalian reproduction, and so closely tied to fitness, there is a great deal of variability in underlying mechanism(s) among major mammalian clades [54, 67, 72, 73]. In fact, a persistent theme in the literature is the lack of suitable animal models for human parturition, and the inadequacy of current rodent models [73]. A second pervasive theme, is the apparent complexity, with human parturition able to be initiated by multiple different pathways, something termed the modular accumulation of physiological systems [73]. Some better characterized mechanisms regulating the timing of human parturition include functional progesterone withdrawal [68, 70, 73–76], the activation of proinflammatory pathways [68–71, 73, 75], and maternally circulating corticotropin-releasing hormone levels [68, 70, 75, 77]. Here, I argue that both of these features (e.g. the interspecific variability and apparent complexity/redundancy) are consistent with a history of maternal-fetal conflict over the control of parturition. I do so by way of analogy.

The placenta has been described as the most variable organ in mammals [3, 78, 79]. Some axes of placental variation include invasiveness (i.e. endotheliochorial, epitheliochorial, hemochorial), interdigitation (i.e. labyrinthine, trabecular, villous), and shape (i.e. cotyledonary, diffuse, discoid, zonary) [80]. With respect to invasiveness, there have been over a dozen transitions among character states [80], and this placental variation appears largely unrelated to ecology [3, 78]. Crespi and Semeniuk [3] amassed considerable data in support of the hypothesis that placental variability instead results from an arms race between maternal and fetal tissue over the physical control of resource provisioning during pregnancy. Specifically, they argue variation in placental characteristics among different groups occurs because of how underlying maternal-fetal conflict plays out, and whether mother or offspring attain relative advantage depends upon lineage-specific factors such as the genetic substrate and available variation [3]. In a somewhat similar vein, Haig [1, 81] has described aspects of fetal-placental hormone production during pregnancy (e.g. the complexity, redundancy, and even wastefulness) as the byproduct of a maternal-fetal arms race over control of the rate of offspring resource provisioning. This is also an area where we see prominent interspecific differences [82]. The timing of parturition may fit a similar mold.

If subject to past struggle over control, then some features of human parturition may be shaped by or exhibit signatures of conflict, including the notable differences between humans and other mammal species [54, 67, 72, 73], multiple pathways of initiation [68, 70, 72, 73, 75], feedback loops with inputs from both mother and fetus [67, 70, 72], and the involvement of dozens of molecules originating from and affecting multiple tissues (e.g. placenta, fetal HPA axis, maternal decidua, maternal brain) [67, 68, 72, 74, 75, 83]. If this view has merit, it may have implications for how the physiological details of parturition are interpreted. Specifically, the above-mentioned features may not make sense solely from an adaptive perspective. Rather than a single, simple, clock-like parturition pathway controlled by either mother or offspring, we instead see redundancy, complexity, and multiple initiating pathways jointly influenced by two genetically distinct individuals—mother and offspring—with partially overlapping fitness optima. As such, parturition timing may be more subject to dysregulation than the timing of other physiological functions that are solely under the control of one individual and therefore not subject to conflict. It has even been suggested that this conflict dynamic (i.e. conflict-induced decanalization) might contribute to the high variance around the timing of birth [84], particularly the high rate of preterm birth (e.g. 11% in the USA, including a substantial fraction with no identifiable cause [85]).

## PRETERM BIRTH

Preterm birth has been described as an adaptive response to an adverse intrauterine environment [86, 87]. But adaptive for whom? It is often assumed that preterm birth allows offspring to escape from a sub-optimal intra-uterine environment [88]. Implicit in this view is that preterm birth is of benefit to both mother and offspring (i.e. making the best of a bad situation). A view less often expressed is that preterm birth may sometimes be beneficial for one party but detrimental for the other—that is, there is a fitness tradeoff between mother and individual offspring. The perspective offered here suggests there may be circumstances in which preterm birth can benefit the mother as a means of reducing investment in the current pregnancy. However, given the potential health costs of being born preterm, offspring are likely to concur only under severe circumstances. In other words, the threshold for initiation of (preterm) parturition is predicted to be lower for mother than offspring. If true, we might expect preterm birth to be more often initiated by the mother. Furthermore, we might expect this initiation when the mother senses the environment is poor, risky, deteriorating, or continuing a pregnancy is likely to be very costly to her health.

A variety of classification schemes have been developed for preterm birth based on putative underlying cause [89–92], and the list of risk factors is extensive [41, 85, 93, 94]. Some well-established causes include infection/inflammation, placental dysfunction/pre-eclampsia, and multiple births [89–92]. Furthermore, some well-established preterm birth risk factors include characteristics of the mother (e.g. less than 18 years of age, low pre-pregnancy BMI), the pregnancy (e.g. short interbirth interval, male fetus), and the environment (e.g. smoking, stress) [41, 85, 93, 94]. In essence, anything that perturbs the pregnancy, thereby making it more costly to the mother, might increase the likelihood of spontaneous preterm birth. Systemic inflammation has been suggested as a pathway by which stress could increase preterm birth risk [53]. It is also worth noting that a large fraction of preterm births (~30% in one recent study) are idiopathic, meaning no known underlying cause can be identified [90]. Interactions between maternal and fetal tissue, particularly at immune recognition loci, may be implicated in some of these [42, 95, 96]. In conclusion, whether preterm birth is considered adaptive, and for whom, may depend upon whether there is an underlying pathological cause, its severity, and the offspring’s stage of development—in other words, the predicted fitness costs and benefits to both mother and offspring. Finally, having a mechanistic understanding of parturition (in terms of physiological processes) won’t change any underlying tradeoff that exists between maternal health and individual offspring fitness. That is, delaying birth through administration of pharmaceuticals (without addressing underlying cause) could entail costs to maternal health. Conversely, initiating birth for maternal convenience, when it is not otherwise medically warranted, may entail costs to the offspring.

## CONCLUSION

**Figure 5:**
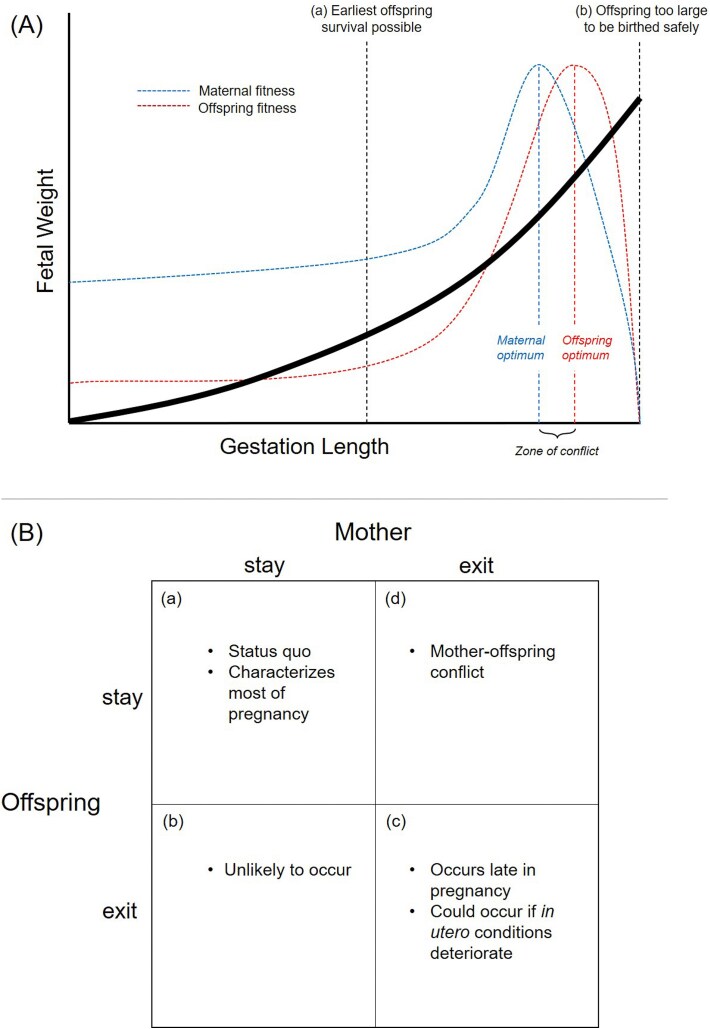
Conceptual models of maternal-fetal conflict and the timing of birth. (A) Fetal weight increases as a function of gestation length, due to resource transfer across the placenta (black line). Birth can theoretically occur at any point along this curve. Below a certain threshold gestation length (a) offspring are too small and underdeveloped to survive postnatally. Above a certain threshold gestation length (b) offspring are too large to be safely birthed. A narrow region exists where the optimal timing of birth differs for mother and offspring (zone of conflict). Maternal and offspring fitness curves are indicated in blue and red, respectively. Note that a mother retains positive fitness prior to the earliest point where offspring survival is possible because her fitness is not entirely dependent upon the current pregnancy. (B) The timing of birth can be modeled with a 2 × 2 contingency table. At any given point during pregnancy, an offspring has two options—attempt to stay *in utero* (stay) or initiate birth (exit). Likewise, a mother has two options—attempt to maintain the pregnancy (stay), or initiate birth (exit). This results in four possible combinations, indicated in boxes (a) through (d). (a) The condition in which the offspring seeks to stay *in utero* longer, and the mother concurs, is characterized by agreement, or no conflict. This could be seen as the normal pregnancy state or status quo, which characterizes most of pregnancy. (b) The condition in which the offspring seeks an exit (i.e. to be born), but the mother seeks for the offspring to stay *in utero*, is characterized by conflict. This situation may be unlikely to occur as it is equivalent to the mother providing more resources than the offspring is willing to accept. (c) The condition in which the offspring seeks to be born, and the mother concurs, is characterized by agreement, or no conflict. Presumably, there is a point late in pregnancy where this occurs, a point at which the offspring has obtained its optimum size (likely beyond the maternal optimum). This situation presumably could also occur if the offspring has reached the point of viability and *in utero* conditions have deteriorated to such an extent that the mother seeks birth to protect her own health, and the offspring also would be better off outside the womb. (d) Finally, the condition in which the offspring seeks to stay *in utero* longer, but the mother seeks to initiate birth, is characterized by conflict. This situation is predicted to occur during a narrow window of time after offspring viability has been reached, but before the offspring has obtained its optimal (large) size. This is the primary subject of this paper.

Birthweight and gestation length are functionally linked. One means by which offspring could obtain larger size at birth is by extending gestation. Abundant evidence indicates gestation length is jointly influenced by both mother and fetus, and I argue that offspring are likely to favor slightly longer gestation than is optimal for the mother. This is predicted to create the necessary conditions for maternal-fetal conflict (Fig. 5). The effect of imprinted genes on gestation length, as seen in imprinting disorders, is consistent with this hypothesis. I have argued that some details of parturition, including variability in underlying mechanism(s) among different mammalian clades (i.e. a lack of conservatism) and the layered complexity and accumulated redundancy of initiating mechanisms observed in humans, may be remnants of a history of maternal-fetal conflict over the control of parturition. I have suggested, somewhat speculatively, that the timing of birth may be subject to dysregulation owing to conflict-generated instabilities (and this may contribute to the high rate of preterm birth). Finally, I have suggested that not all instances of preterm birth are adaptive for both parties, but could instead sometimes involve a tradeoff between maternal and offspring fitness. Although this paper emphasizes maternal-fetal conflict (as this may be underappreciated with respect to gestation length) the converse is that producing healthy offspring requires a great deal of cooperation, and conflict will be tempered by the mutual interdependence and genetic relatedness of mother and offspring.

## Data Availability

The data used to construct Figs 1–3 is publicly available: USA Vital Statistics Natality Birth Data from the National Bureau of Economic Research (https://www.nber.org/research/data/vital-statistics-natality-birth-data).
